# Integrating the exposome and one health approach to national health surveillance: an opportunity for Latin American countries in health preventive management

**DOI:** 10.3389/fpubh.2024.1376609

**Published:** 2024-08-14

**Authors:** Patricia Matus, Cinthya Urquidi, Marcela Cárcamo, Veronica Vidal

**Affiliations:** Department of Epidemiology and Health Studies, Universidad de Los Andes, Santiago, Chile

**Keywords:** exposome, One Health, surveillance, double burden, Latin American

## Abstract

The exposome approach, emphasizing lifelong environmental exposures, is a holistic framework exploring the intricate interplay between genetics and the environment in shaping health outcomes. Complementing this, the one health approach recognizes the interconnectedness of human and ecological health within a shared ecosystem, extending to planetary health, which encompasses the entire planet. Integrating Disease Surveillance Systems with exposome, one health, and planetary health signifies a paradigm shift in health management, fostering a comprehensive public health framework. This publication advocates for combining traditional health surveillance with exposome and one health/planetary health approach, proposing a three-step approach: ecological analysis, territorial intervention in identified issues, and an analytical phase for assessing interventions. Particularly relevant for Latin American countries facing a double burden of diseases, integrating the exposome into traditional health surveillance proves cost-effective by leveraging existing data and environmental measurements. In conclusion, the integration of exposome and one health approaches into traditional health surveillance presents a robust framework for monitoring population health, especially in regions like Latin America with complex health challenges. This innovative approach enables tailored interventions, disease outbreak predictions, and a holistic understanding of the intricate links between human health and the environment, offering substantial benefits for public health and disease prevention despite existing challenges.

## 1 Introduction

Identifying external factors that trigger or contribute to the onset of diseases has been a central goal in epidemiology and public health. While the biological approach has primarily concentrated on individual risk factors and genetic predispositions, the shortcomings of explaining diseases solely through genetics have become evident. Despite the multitude of genome-disease studies conducted, only a small proportion of diseases can be solely attributed to genetic factors (1). On the other hand, current research methodologies face limitations in capturing the complete spectrum of environmental exposures. Considering this, innovative approaches are crucial to assess the cumulative effects of multiple exposures, thus bolstering the case for a more holistic understanding (2).

In public health, the prevailing emphasis on individual risk continues to overshadow the significance of a population-based approach and its determinants (3). Recent European developments, such as the Athlete project, the EPHOR project, the Helix project, and the Public Health Exposome Perspective in the USA, are embracing a holistic strategy based on the Exposome theory. Most of these initiatives focus on European populations with varied geographical scopes or specific diseases and health outcomes, including early-life development, cardiovascular and metabolic diseases, chronic respiratory diseases, or major chronic diseases. These endeavors delve into the interconnectedness of environmental exposures and human signals of diseases within specific populations or worker cohorts (4–8).

Athlete project for example developed an approach to extract and summarize the existing evidence about the effects of environmental factors on cardiometabolic, neurodevelopmental, respiratory, and other birth and child health outcomes. They found 16 environmental exposures with great levels of evidence (organic compounds, metal, temperature, and green space, among others) (9).

As we advance, the Public Health Exposome proposes the use of Big Data to Knowledge, an exposome paradigm, and computational, Bayesian, and spatial-temporal approaches offer tremendous promise in identifying biomarkers of subclinical, cardiovascular/cardiometabolic disease, informing novel diagnostic and treatment options, and informing public and environmental health policy. Such studies will determine if biomarkers of exposure, effect, or disease susceptibility might enhance the prediction of future disease trajectories and provide earlier opportunities for intervention (10).

## 2 The exposome approach and future directions

The exposome concept, introduced by Wild (11), underscores the importance of examining cumulative exposure to non-genetic factors in epidemiological studies, particularly in cancer research. Wild posited that the exposome, complementing the genome, provides a comprehensive account of individuals' lifelong exposure history (11).

In 2012, Wild further delineated the exposome into three categories: the internal exposome, specific external exposome, and general external exposome. The internal exposome involves biological factors influencing individual health outcomes, while the external exposome encompasses lifestyle, occupational, and environmental factors. The specific external exposome pertains to direct damage from physical or chemical agents, such as radiation, infectious agents, chemical contaminants, smoking, alcohol consumption, occupation, and medical interventions measured through individual exposures typically obtained from questionnaires. On the other hand, the general external exposome includes measurable population-level exposures, such as climate, social capital, education, financial status, mental and psychological stress, and the urban-rural environment. These factors contribute to an indirect mechanism of damage, involving the stimulation of the hypothalamus-pituitary axis, which, in chronic situations, triggers systemic pathophysiological mechanisms.

Beulens et al. present the following model as an example of the exposome approach to understanding the causality of Type II Diabetes Mellitus. High urbanization (General External Exposome) is associated with exposure to atmospheric pollution by PM2.5 (External Specific Exposome), which, in turn, is linked to alterations in endothelial function, inflammation, and insulin resistance (Internal Exposome) (12).

Currently, it is acknowledged that environmental factors account for 70%−90% of the risk of illness, a concept extensively utilized in studies on the causality of major non-communicable chronic diseases (13–17).

The exposome framework is pivotal for understanding individual exposures, a crucial aspect for both One Health and Planetary Health. One Health recognizes the interconnectedness of human, animal, and environmental health, fostering collaboration across disciplines like medicine, veterinary science, ecology, and public health. The exposome is integral to One Health, as environmental exposures impact not only human health but also the health of animals and the broader ecosystem (18).

Planetary Health broadens its scope beyond individual and species wellbeing to encompass the overall health of our planet. This field investigates how changes in the environment, such as climate change, loss of biodiversity, and pollution, impact the health of both human populations and ecosystems worldwide. The exposome, which encompasses all external factors influencing health, is integral to understanding how human activities affect planetary health. Integrating exposome principles with One Health and Planetary Health is crucial for devising comprehensive strategies to tackle the complex challenges where human, animal, and environmental health intersect (19, 20).

Research with this integrative approach also has practical importance as it facilitates the translation of basic, molecular knowledge to decision-making in public health. An example of the latter are interventions in childhood obesity and phthalate exposure, Vineis et al. suggest that an exposome-based approach can help strengthen causal reasoning and support restrictive intake of UPF policy in children for avoid obesity. Gerofke et al. found that result indicators proved to be suitable for demonstrating the effectiveness of policy measures for phthalate. They showed similar exposure for boys and girls, indicating that there is no need for gender focused interventions and/or no indication of sex-specific exposure patterns (21, 22).

## 3 Expanding exposome studies to infectious disease and disasters

While exposome research has primarily focused on chronic non-communicable diseases, recent attention has broadened to include infectious diseases such as COVID-19 and the impact of disasters on public health due to the COVID-19 pandemic and the climate crisis (23, 24). The consideration of infectious diseases and the impact of disasters on public health is particularly challenging and potentially intriguing for developing countries or emerging economies. Unlike high income countries, they must contend with the double burden of diseases stemming from unsanitary conditions of underdevelopment and the burden of chronic diseases associated with a pandemic.

Consequently, these novel applications of the exposome approach may facilitate the adoption of measures to promote health and prevent diseases in specific populations directly or potentially exposed to such environmental stressors. Measuring regional stressors will enable the correction of conditions posing an unacceptable risk to the population. Therefore, quantifying patients will not be necessary, as is the case with adopting measures against clusters. To initiate the establishment of sanitary measures, evidence of harmful exposure will suffice for their development.

## 4 Exposome approach for Latin American health issues

For Latin American countries and low-income countries grappling with the double burden of diseases, characterized by the simultaneous presence of infectious and non-communicable diseases (25), the challenges are multifaceted. Rapid demographic and epidemiological transitions in Latin American countries have led to the persistent prevalence of infectious diseases like malaria, tuberculosis, and diarrheal diseases, alongside a rising incidence of non-communicable diseases such as cardiovascular diseases, diabetes, and cancer. Contributing factors to this dual burden include urbanization, economic development, and an aging population. The shift from rural to urban living is associated with lifestyle changes, including sedentary behavior and unhealthy diets. Additionally, improved economic conditions have resulted in changes in dietary habits and increased tobacco and alcohol consumption. These factors, coupled with a longer life expectancy, contribute to the growing prevalence of age-related diseases.

The phenomenon of the double burden poses significant challenges to public health systems, requiring them to simultaneously address the prevention and control of infectious diseases while managing the escalating burden of non-communicable diseases (26, 27). Moreover, health management must contend with heterogeneous disease patterns within countries (28).

The exposome approach has great potential to address health problems in Latin America. While initiatives in high-income countries use large cohort studies that integrate social and environmental data with individual biological measurements, these methodologies are unlikely to be feasible in Latin American regions or other medium and low-income countries due to economic costs and limitations operational difficulties.

A more cost-effective and appropriate mechanism involves leveraging traditional health surveillance. This hypothesis is reflected in a research proposal, one of whose purposes is to test the ability to manage and analyze large databases incorporating disease patterns, environmental stress factors, and social determinants of health in population health evaluations. This will be achieved using statistics from national-level sources, including ministries of health, social services, education, and economics, in a Latin American country. The experience gained from its future implementation will allow for the collection of best practices, enhancing its adaptation to other realities in Latin America (29).

Therefore, the purpose of this article is to propose a broader perspective on the exposome/one health approaches, establishing a conceptual framework that integrates them with traditional health surveillance. This integration aims to monitor population health conditions, and environmental stressors and design strategies for specific sub-national territories to effectively address the challenges posed by the double burden of diseases.

## 5 Integrated health system and external exposome for preventive management

Traditional disease surveillance entails the ongoing monitoring and analysis of data concerning the occurrence and dissemination of diseases within a population. This encompasses information on reported cases, demographic data, clinical characteristics, and the geographic distribution of diseases. Currently, the International Health Regulations of 2005 define surveillance as “the systematic collection, comparison, and analysis of data for public health purposes, and the timely dissemination, assessment, and response, as appropriate” (30).

For instance, Chile has implemented a national system for the surveillance of communicable diseases since 1990 (31), and more recently, legislation has established the creation of the national cancer registry (32). To analyze non-communicable diseases, the country incorporates mortality counts, hospitalizations, and/or emergency care data into its systems to objectify temporal trends and spatial patterns, identifying deviations from expected national patterns. These deviations can justify more precise public health strategies tailored to subnational needs. Additionally, the country operates a national air quality network to monitor air pollution, gathers information on water quality (33), and tracks socioeconomic indicators (34).

Access to the management of large amounts of systematically recorded information, statistical analysis methods, forecasting capabilities, and report generation facilitates the incorporation of agents, conditions, and morbidities into integrated systems. These systems account not only for traditional communicable diseases but also for non-communicable diseases and conditions that cause the most significant disease burden in the country and subnational territories.

**Figure 1:**
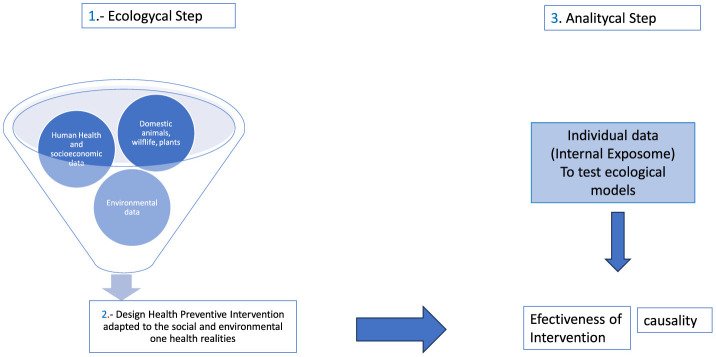
Integrated information system for preventive disease management.

Epidemiology and statistics, or the science of data analysis as it is currently known, are the primary tools for converting data into information for decision-making in disease prevention. For the preventive management of diseases, we propose integrating data and information by developing three levels of analysis, sequentially in operational complexity: an ecological analysis of information, defining territories for specific preventive interventions, and subsequently, a longitudinal analysis of a sample of individuals to answer hypotheses generated during the ecological-level analysis (Figure 1).

During the ecological phase of analysis, relationships are established between specific components of the external exposome and one health components, selecting particular territories. This allows the design of intervention measures adapted to the social and environmental realities of each subdivision of the territory. In this first phase, associations between the external exposome and selected diseases from the health surveillance system are measured through ecological statistical methods. Territories with anomalies and their associated factors are identified, and in the second phase, intervention measures are planned to control the identified problems.

The third phase involves refining the statistical models by incorporating individual measurements from cohorts of individuals in the established territories for conditions that pose a public health problem. These designs allow the evaluation of causality and the effectiveness of proposed interventions.

## 6 Variables incorporated in the integrated system

Our understanding of diseases and conditions linked to environmental factors has seen remarkable growth, driven by a surge in systematic reviews and meta-analyses that quantify the strength of association and attributable risk for each exposure (35). Additionally, recent publications have identified new diseases associated with known pollutants (36).

Table 1 highlights some components of the external exposome measured in Chile, encompassing air and water pollutants, noise, meteorological variables, socioeconomic and social indicators. The quantification of exposure agents in column 1 relies on environmental measurements, including air quality readings from the National Air Quality Network, as well as individual and aggregate measurements obtained through questionnaires from various ministries. Additionally, it facilitates the computation of indices, such as the Environmental Justice Index (EJ), enabling targeted health promotion and prevention interventions in the most vulnerable populations (37). It's crucial to note that maternal-child diseases, with their escalating burden of chronic illnesses, should also be addressed from an ecological and life course perspective (38).

**Table 1:** Chilean external exposome and health variables to incorporate into the integrated system.

**External exposome**	**Health status**
Physical environment: **Air component (specific external exposome):** • Primary pollutants: particulate matter; carbon monoxide, sulfur and nitrogen dioxide; ozone • Hazardous pollutants: lead, arsenic, benzene • Temperature and light • Environmental noise: day and night **Water component (specific external exposome):** • Composition of wastewater • Biological agents: bacteria, viruses, and protozoa • Harmful algal blooms • Heavy metals: arsenic, mercury, lead, and cadmium • Organic compounds: benzene, pesticides, trichloroethylene (TCE) **Soil Component (specific external exposome):** • Heavy metals • Pesticides Stable global environment (specific external exposome): • UV radiation • Heat and cold waves • Floods (extreme climate events) **Built and social environment (general external exposome):** • Domestic violence • Criminal violence/drug trafficking • Communal economic income • Community education • Access to health care • Access to nature (% green areas) **Grouped behaviors (general external exposome):** • Food: diet • Drugs • Alcohol • Tobacco • Marijuana • Cocaine	•Obesity • Cardiovascular risk • Sedentary lifestyle • **Mortality:** Cancers Suicide Dementia Stroke Cardiovascular diseases: AMI Respiratory diseases: Asthma—COPD • **Morbidity:** Cancers (National Registry) Hospital discharges Emergency consultations Cognitive Deficit in children and adults Preterm delivery

The information typically originates from multiple institutions, many of which operate outside the health sector. Consequently, utilizing this data poses a significant challenge in terms of standardization and integration to derive actionable insights. This process involves geospatial integration, data fusion, analysis of health and environmental datasets, and the implementation of early warning systems. Integrating geospatial and temporal data from both health and environmental sources is a key mechanism for mapping disease patterns against environmental factors. This provides valuable insights into spatial correlations and helps identify new potential environmental risk factors (39). Moreover, advanced analytical techniques, including machine learning algorithms, can be deployed to uncover patterns, correlations, and trends that may remain obscured when examining omics, health, or environmental data in isolation. By combining health and environmental data, an integrated system can predict disease outbreaks determined by environmental conditions. For instance, it can track the prevalence of waterborne diseases during periods of heavy rainfall or high temperatures (40–42).

Assessing the internal exposome presents substantial challenges that demand meticulous consideration in the subsequent stages of research. These complexities are particularly pronounced when dealing with variables originating from individuals who have a right to privacy regarding the management of their results. The sources of these variables can introduce complications and risks, especially in instances where invasive sampling methods, such as venipuncture, are employed. The intricate balance between the imperative to collect accurate data and the ethical and practical concerns associated with privacy underscores the intricacy of measuring the internal exposome.

Specific or general exposome stressors induce biological changes, manifesting as low molecular weight compounds measurable through high-resolution mass spectrophotometry, thereby constituting the metabolome. These internal exposome molecules signify exogenous chemicals or internal products influenced by the external exposome, allowing for measurement, and tracking in individuals (13, 43).

Numerous studies are dedicated to elucidating the biochemical links between environmental factors and diseases. For instance, the literature highlights that exposure to environmental noise and mental stress triggers a pathophysiological response mediated by the hypothalamic-pituitary system, leading to elevated cortisol levels, subsequent inflammation, and oxidative stress. Similarly, exposure to heavy metals, airborne particulate matter, and other toxins directly induces oxidative stress and tissue inflammation. In these instances, both inflammation and oxidative stress play pivotal roles in metabolic and cardiovascular diseases, cancers, and neurodegenerative diseases (44).

Various methods are employed to measure compounds or damage mechanisms, with omics technologies and micro-RNAs being of particular interest as potential biomarkers for objectively and precisely assessing population exposure (45, 46). For example, the neutrophil-lymphocyte ratio (NLR) emerges as a cost-effective, straightforward, rapidly responsive, and easily obtainable parameter for evaluating stress and inflammation. While the NLR exhibits high sensitivity and low specificity, making it applicable against exposure to multiple factors of the external exposome and/or mixtures of contaminants (47).

## 7 Discussion

Currently, there is a discernible trend in modeling development that places a significant emphasis on environmental components. However, a notable drawback is the frequent absence of other crucial contextual variables in many of these models. This deficiency impedes the comprehensive management of public health issues. As an illustration, within the domain of environmental health management, conceptual frameworks have been primarily devised to facilitate the creation of early warning systems addressing episodes of heightened atmospheric pollution (48). An impactful development stemming from the COVID-19 pandemic is the introduction of wastewater-based epidemiology (WBE), a model that incorporates health data (49).

Moreover, a proposal tailored for African countries outlines four phases for establishing a more holistic system: (a) Establishment of environmental fingerprints, (b) Socioeconomic fingerprints, (c) Statistics and modeling, and (d) Information systems (50).

The concept of environmental fingerprints involves defining specific external exposome variables pertinent to public health, as detailed previously (see Table 1). The socioeconomic fingerprint corresponds to variables within the external general exposome listed in the same column. Statistics and modeling entail identifying association structures between external exposome variables and diseases or conditions with a higher prevalence than expected in territorial subgroups or human subgroups (children, the older adult, types of workers, etc.).

This perspective article aims to present an innovative framework for monitoring population health by integrating exposome and One Health approaches with traditional health surveillance. While the USA and European countries have established exposome evaluation projects that track pregnancies and children, integrating exposures and assessing cumulative components through cohort designs, we propose initial steps for Latin American countries using more affordable ecological designs.

Ecological designs involve data integration, providing holistic insights into population health by recognizing the interconnectedness between human health and the physical and social environment (51). This integration enhances predictive capabilities, enabling the development of models anticipating how environmental factors contribute to disease spread. Authorities can then implement preventive measures more effectively, allocating resources based on areas with a higher risk during disease outbreaks linked to environmental factors. Additionally, data integration contributes to community engagement by offering a clearer understanding of environmental factors affecting health, empowering communities to take proactive measures to mitigate risks.

Despite its potential to improve health monitoring and develop locally relevant initiatives, data integration faces challenges and considerations. Key factors include data standardization, privacy and ethical concerns, and interdisciplinary collaborations. Harmonizing diverse datasets for meaningful integration requires careful consideration, while safeguarding sensitive health data alongside environmental information raises privacy and ethical concerns. Implementing measures to change lifestyle becomes essential for preventing diseases (52). Ultimately, successful integration demands collaboration between health professionals, environmental scientists, data analysts, and policymakers, underscoring the importance of interdisciplinary teams for effective system development and maintenance. Specifically, developing data standardization and privacy strategies is crucial to overcoming these challenges.

In a systematic review of ethical aspects of exposome research, five ethical themes are identified for discussion: the goals of exposome research, its standards, its tools, how it relates to study participants, and the consequences of its products. Additionally, the actionability of its findings, the relevance of epidemiological or clinical norms to exposome research, and the significance and implications of bias are three key areas of exposome research that require ethical reflection (53).

Interdisciplinary collaborations are essential for advancing exposome research, leveraging diverse expertise to tackle complex health and environmental challenges. However, fostering such collaborations presents its own set of challenges, including communication barriers, divergent methodologies, and institutional silos. Despite these obstacles, successful examples, as mentioned earlier, demonstrate the transformative potential of interdisciplinary efforts in exposome research.

To foster interdisciplinary collaborations, proactive strategies are essential. Establishing clear communication channels, promoting mutual respect, and creating opportunities for knowledge exchange are fundamental. Additionally, cultivating a shared understanding of research goals and methodologies can bridge disciplinary divides and foster synergy among teams.

Challenges in interdisciplinary exposome research include navigating differing disciplinary cultures, addressing power differentials, and ensuring equitable distribution of resources and credit. Overcoming these challenges requires patience, flexibility, and a commitment to inclusivity.

The National Institute of Environmental Health Sciences (NIEHS) proposes that the first step to tackling these issues is to establish a community of practice to define a framework for exposome research, develop guiding principles and best practices, and leverage ongoing efforts and existing resources for operationalizing exposomics (54).

The cost implications of integrated surveillance systems are significant, encompassing the expenses of advanced technologies, software, hardware, and the training required for personnel to effectively manage and utilize these systems. Investment in infrastructure for data storage, processing, and real-time analysis further adds to the financial burden. Additionally, the multisectoral approach necessary for developing these systems introduces coordination challenges, as it requires collaboration among diverse sectors such as public health, law enforcement, information technology, and environmental government agencies. Harmonizing policies, data sharing protocols, and operational procedures across these sectors can be complex and time-consuming. Effective communication and the alignment of objectives are crucial, but differences in priorities, resources, and expertise can hinder seamless cooperation. Hence, while integrated surveillance systems hold the promise of enhanced security and efficiency, their development demands substantial financial resources and a concerted effort to overcome interdisciplinary barriers.

In conclusion, fostering interdisciplinary collaborations requires deliberate effort, but the rewards are substantial. By overcoming challenges and building on successful examples, interdisciplinary teams can drive innovation, advance knowledge, and ultimately improve human health and wellbeing.

## Data Availability

The original contributions presented in the study are included in the article/supplementary material, further inquiries can be directed to the corresponding author.
